# Supplementary figures and images for: Relationship Between Systemic Inflammatory Response Exponents, Levels of ADAM10, ADAM17 Proteins and Selected Clinical Parameters in Patients with Colorectal Cancer: Original Research Study
Source: Int J Mol Sci. 2025 Jan 27;26(3):1104. doi: 10.3390/ijms26031104 (PMC11817235; doi:10.3390/ijms26031104)

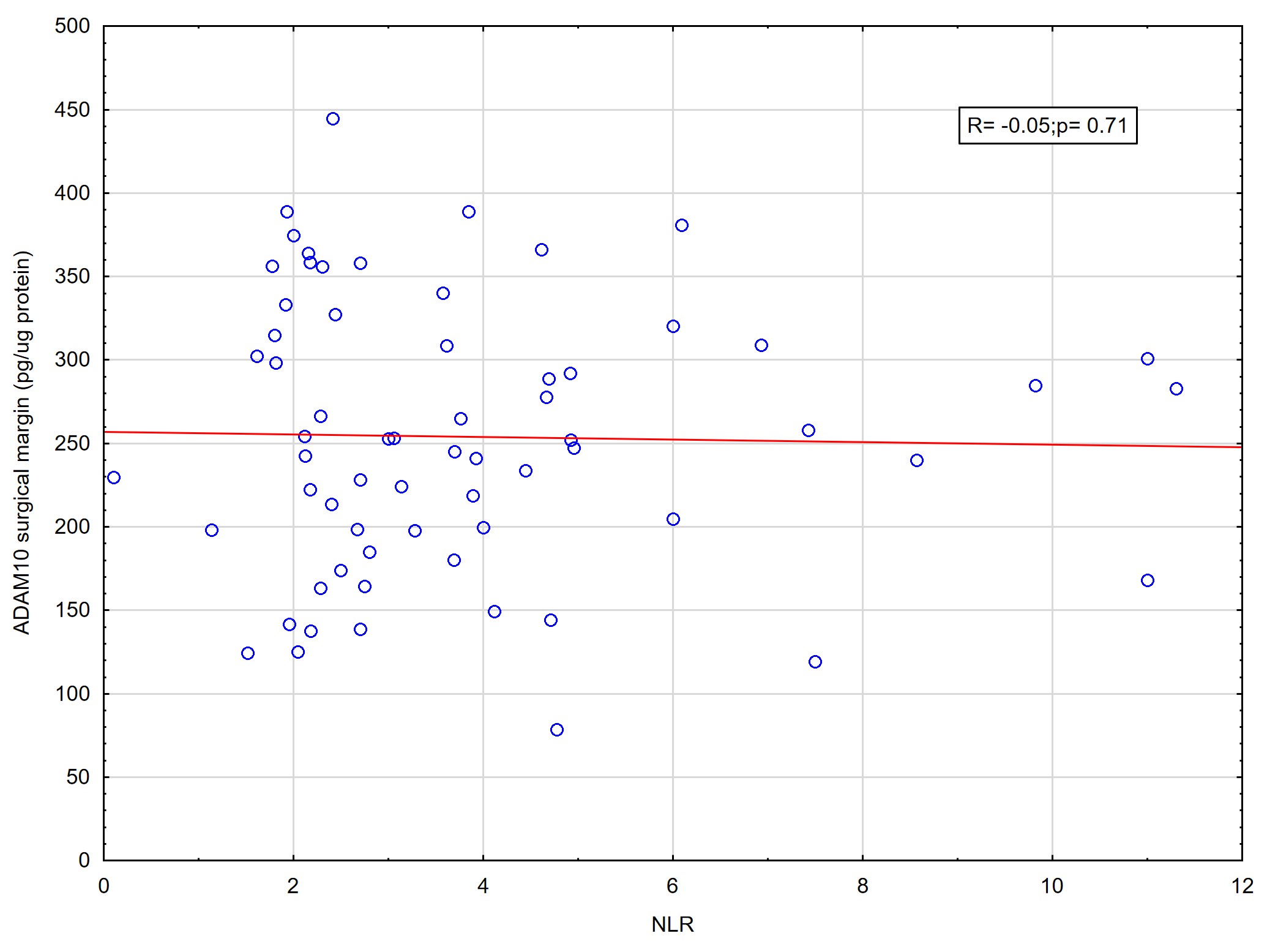

Supplement: Supplementary file 1 [file ijms-26-01104-s001.zip › Figure S3. Correlation between NLR and ADAM10 concentration in surgical margin tissue..jpg]

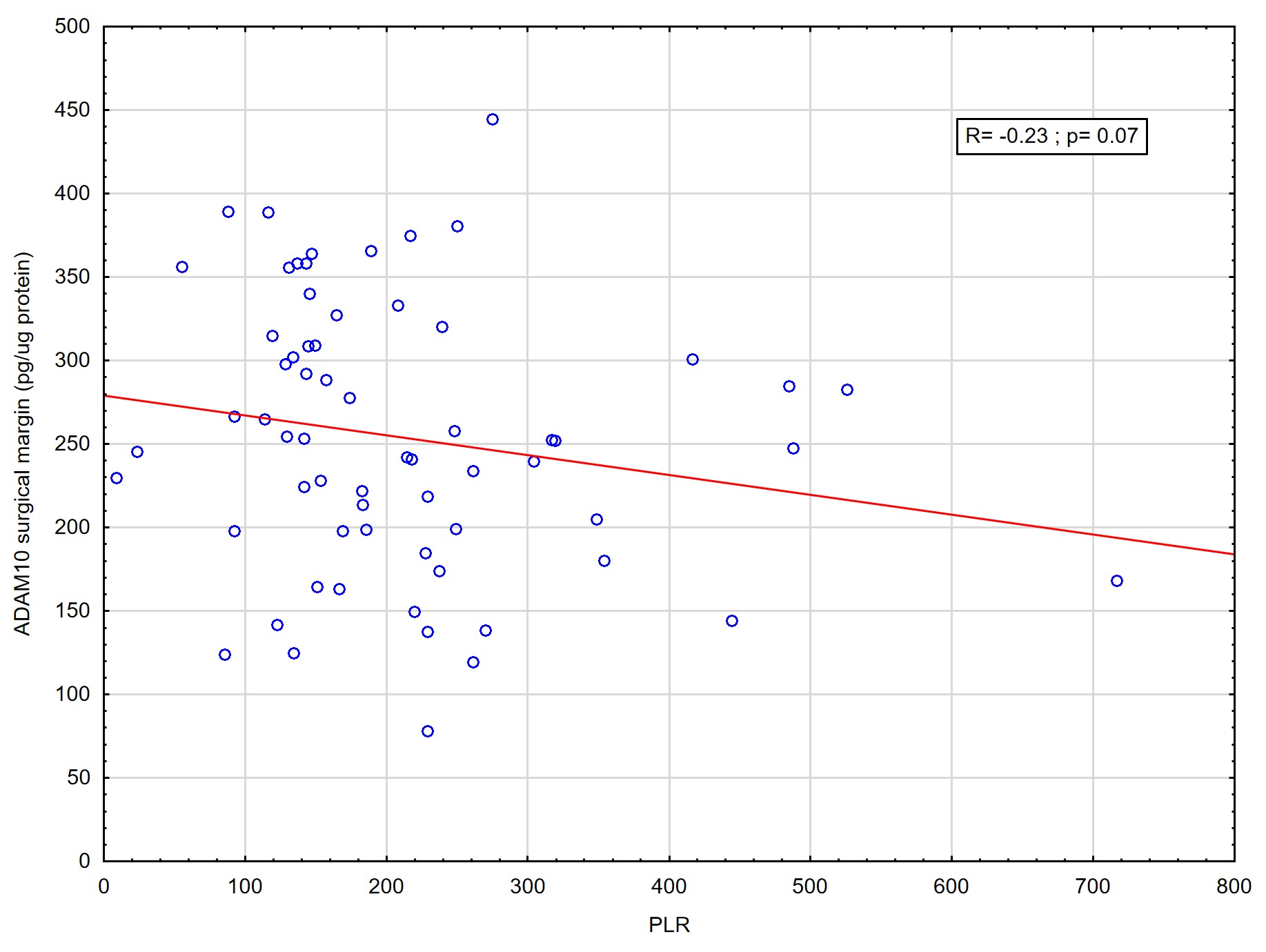

Supplement: Supplementary file 1 [file ijms-26-01104-s001.zip › Figure S4. Correlation between PLR and ADAM10 concentration in surgical margin tissue..jpg]

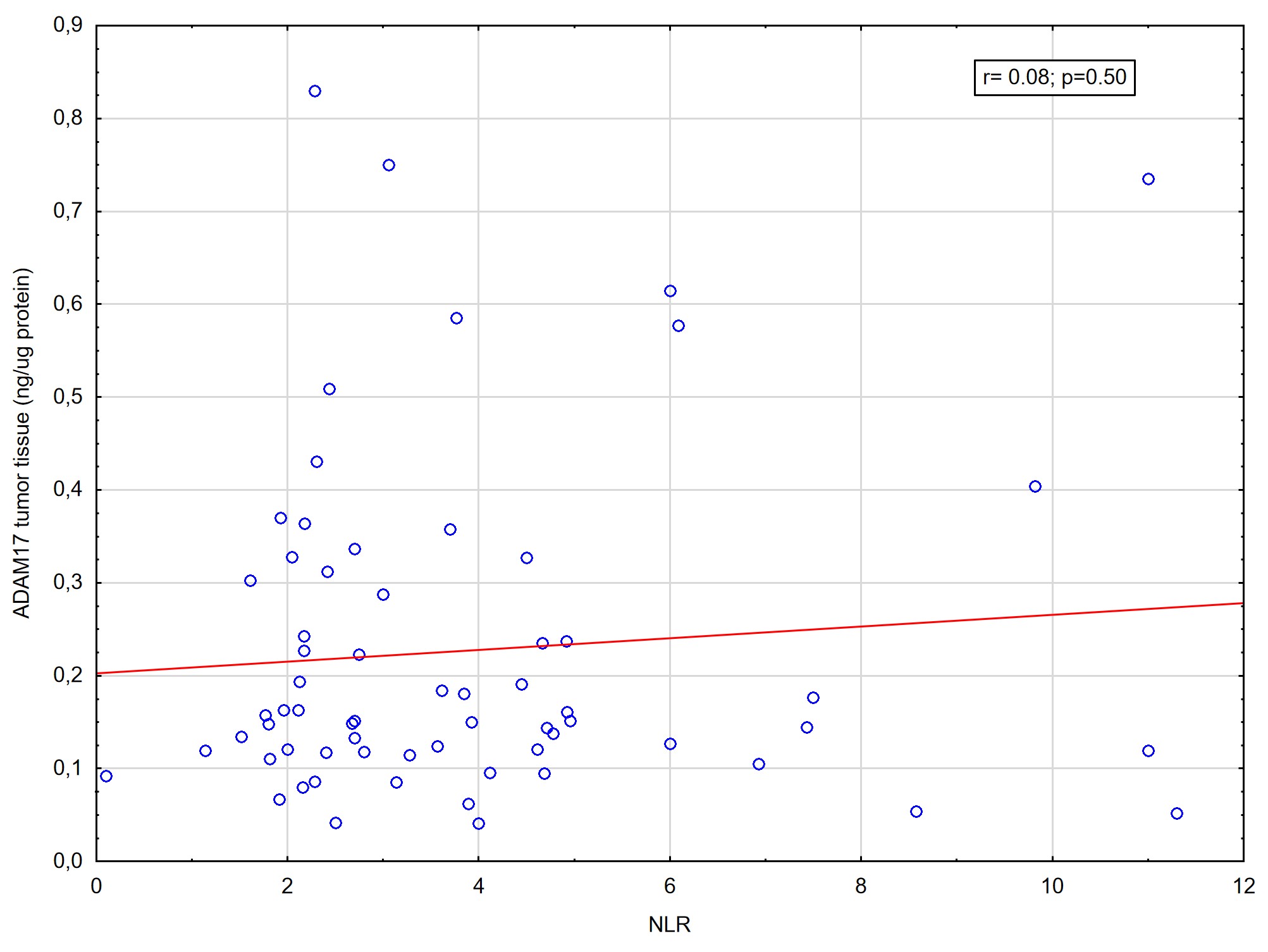

Supplement: Supplementary file 1 [file ijms-26-01104-s001.zip › Figure S5. Correlation between NLR and ADAM17 concentration in tumor tissue..jpg]

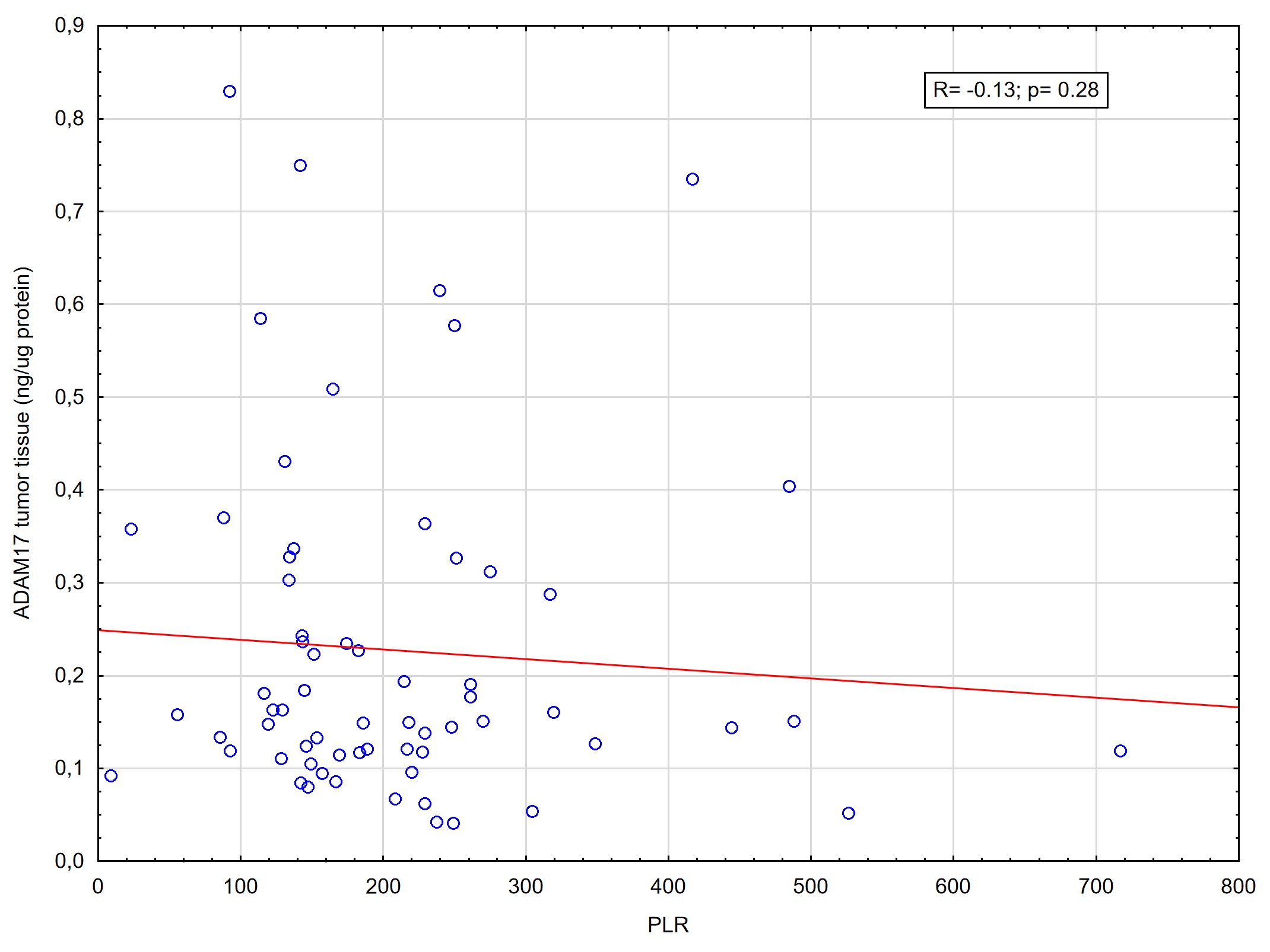

Supplement: Supplementary file 1 [file ijms-26-01104-s001.zip › Figure S6. Correlation between PLR and ADAM17 concentration in tumor tissue..jpg]

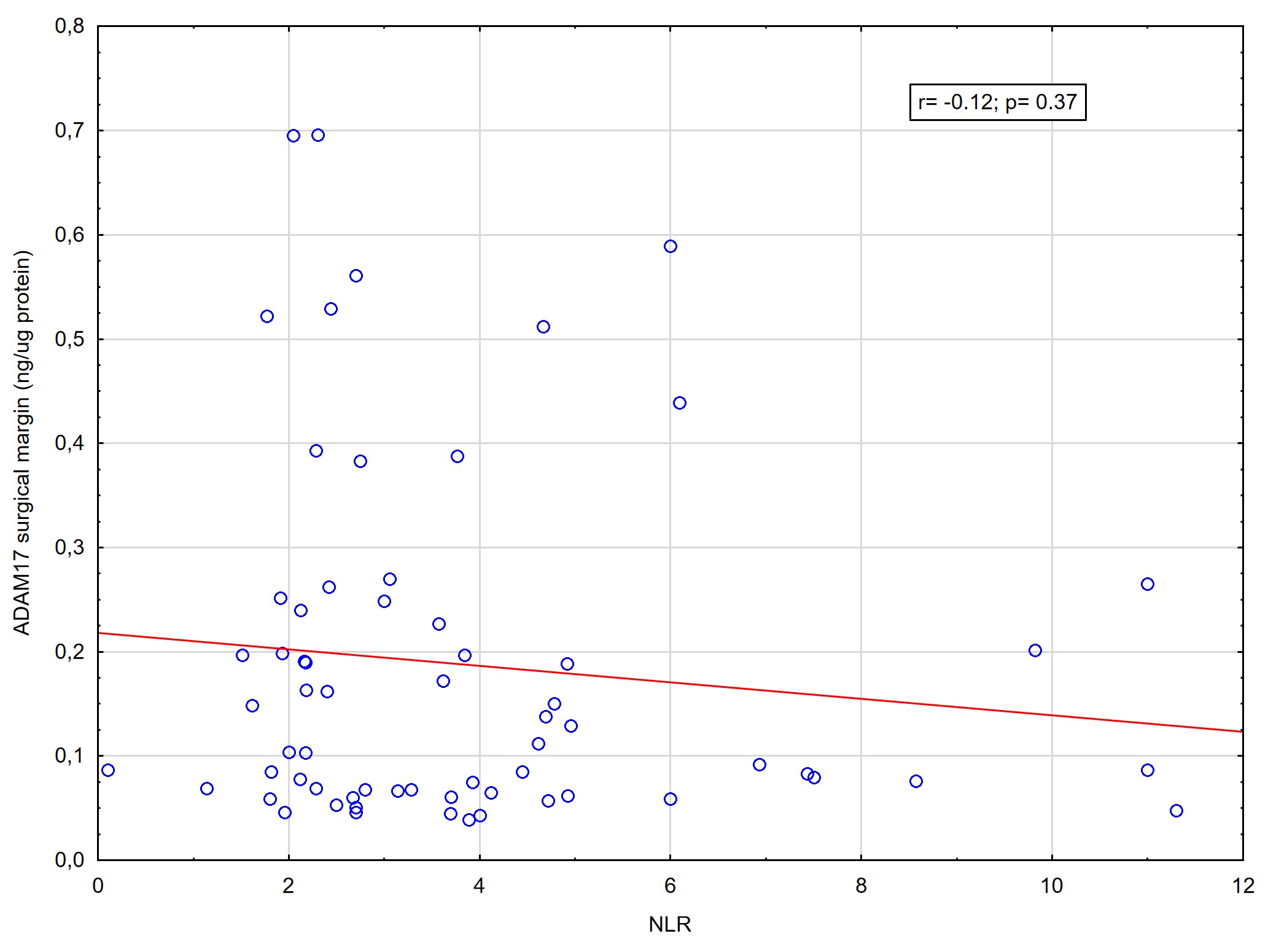

Supplement: Supplementary file 1 [file ijms-26-01104-s001.zip › Figure S7. Correlation between NLR and ADAM17 concentration in surgical margin tissue..jpg]

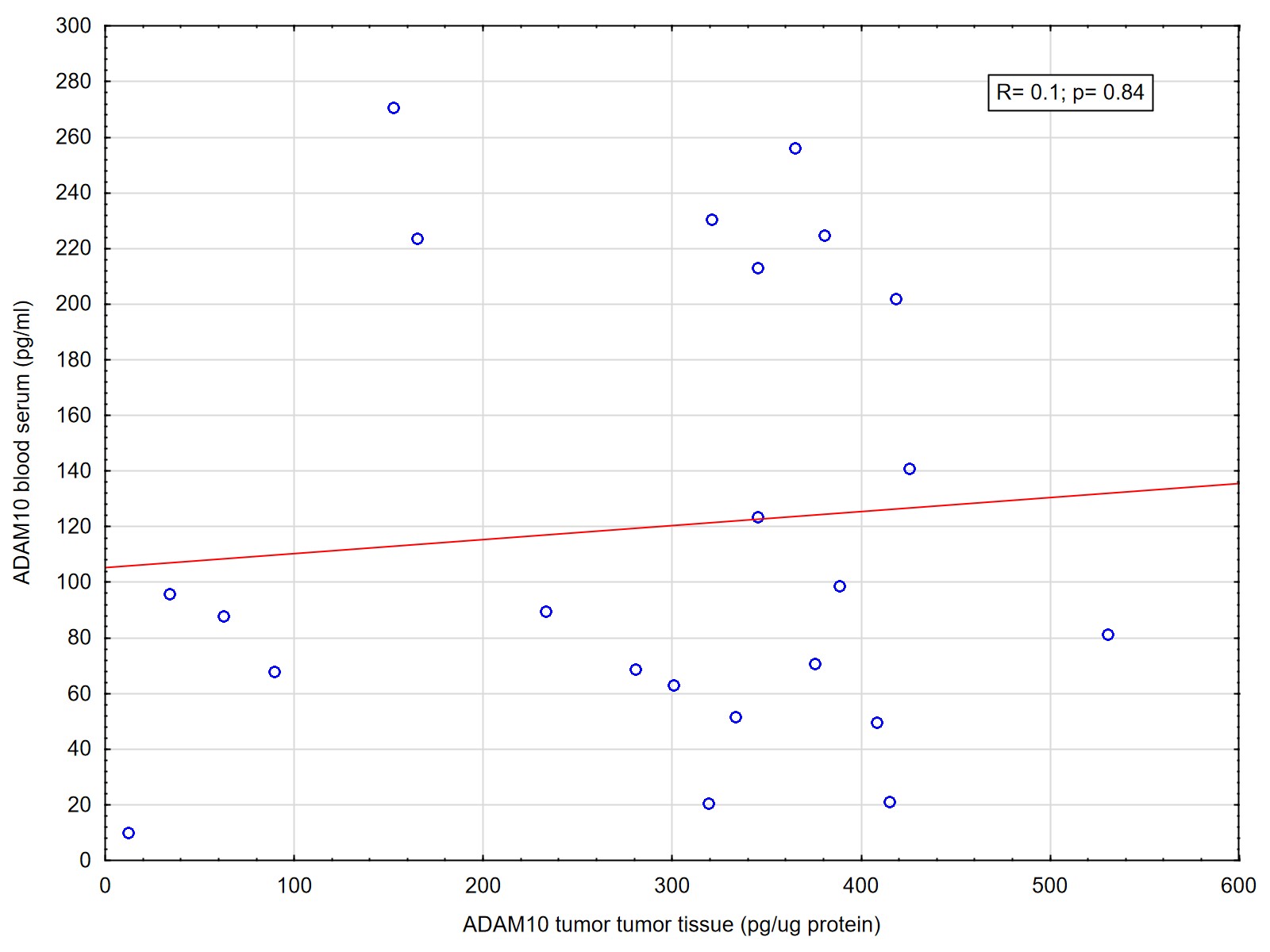

Supplement: Supplementary file 1 [file ijms-26-01104-s001.zip › Figure S8. Correlation between ADAM10 concentration in tumor tissue and ADAM10 concentration in blood serum..jpg]

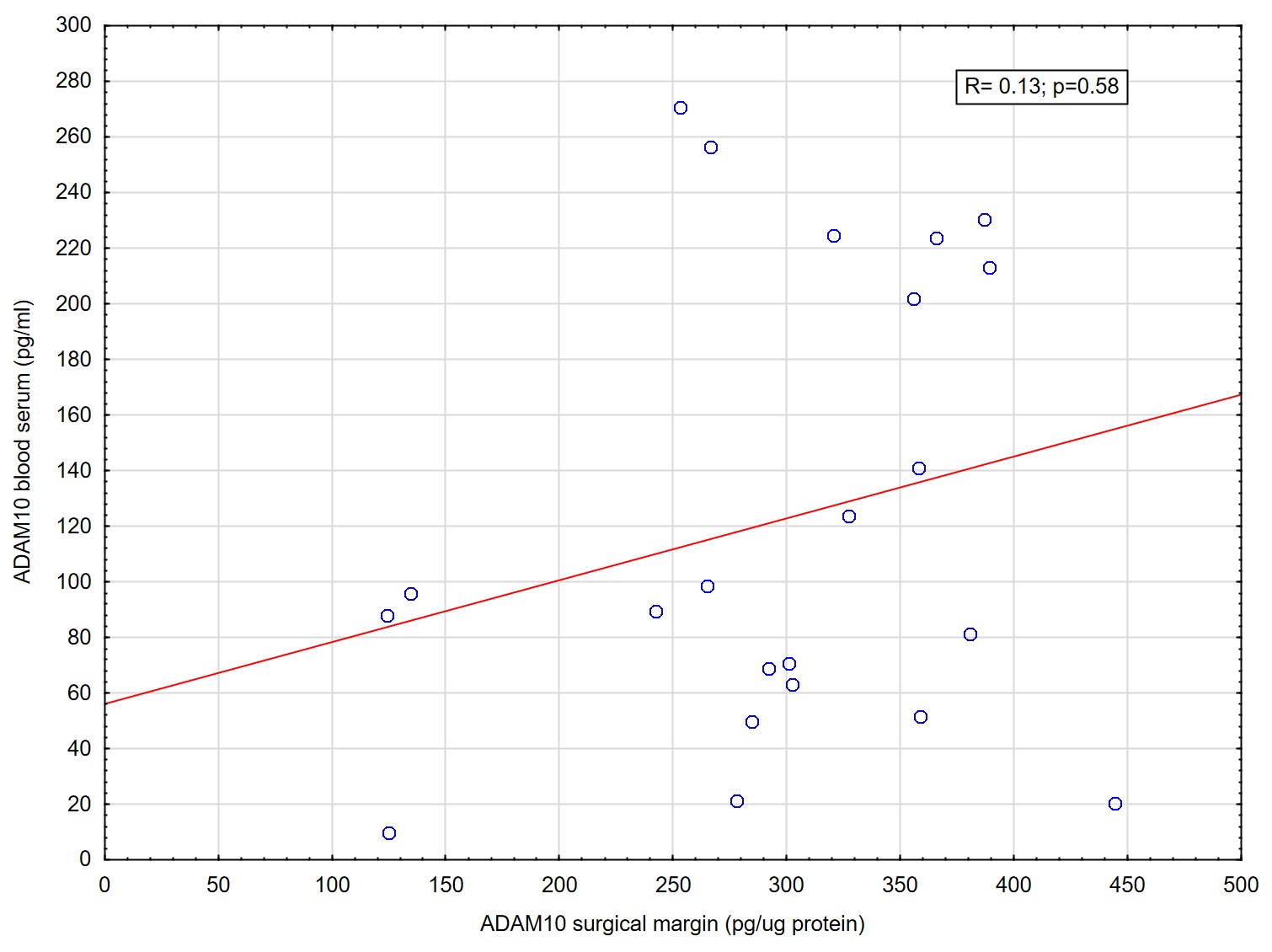

Supplement: Supplementary file 1 [file ijms-26-01104-s001.zip › Figure S9. Correlation between ADAM10 concentration in surgical margin tissue and ADAM10 concentration in blood serum..jpg]

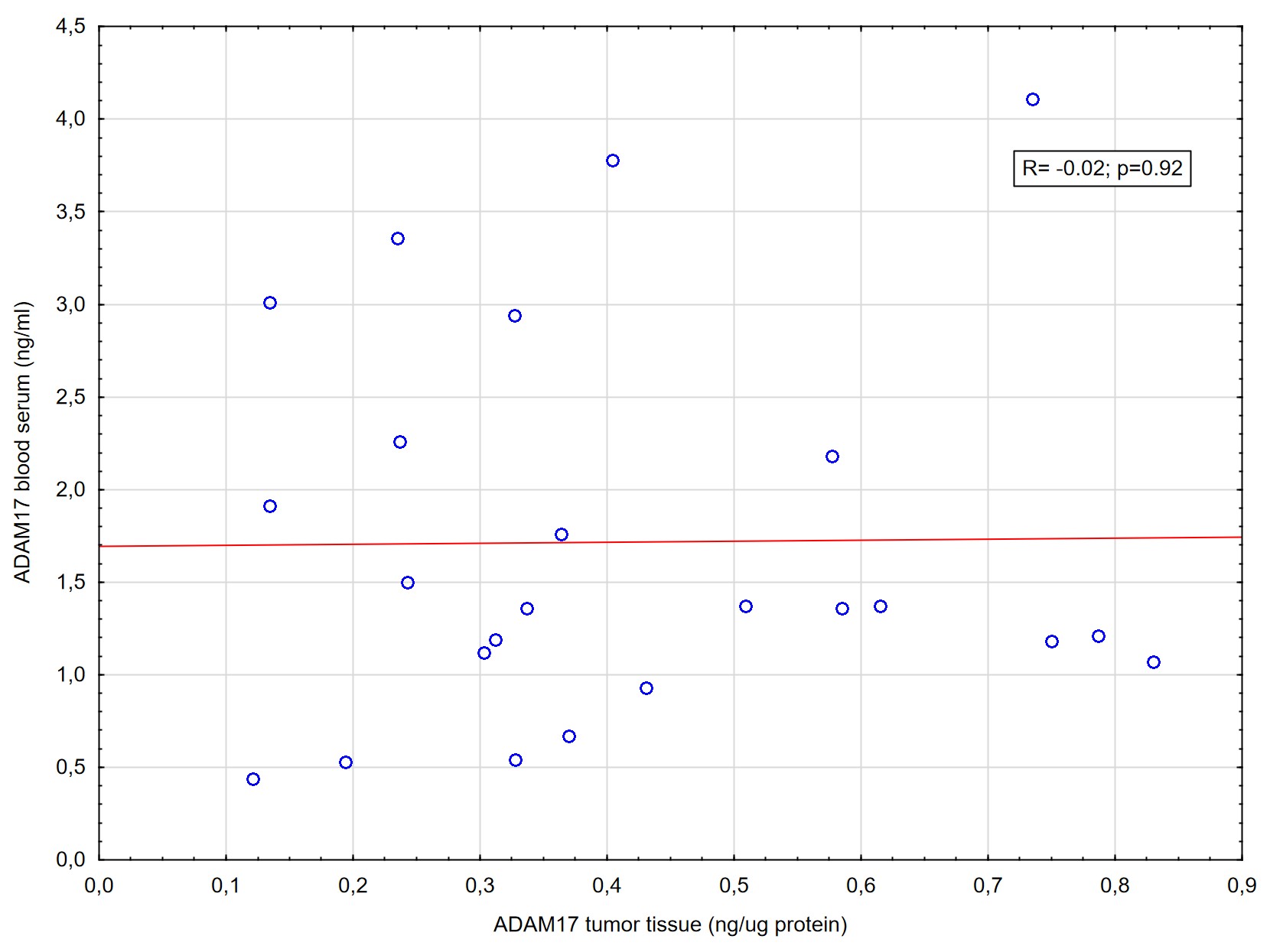

Supplement: Supplementary file 1 [file ijms-26-01104-s001.zip › Figure S10. Correlation between ADAM17 concentration in tumor tissue and ADAM17 concen-tration in blood serum..jpg]

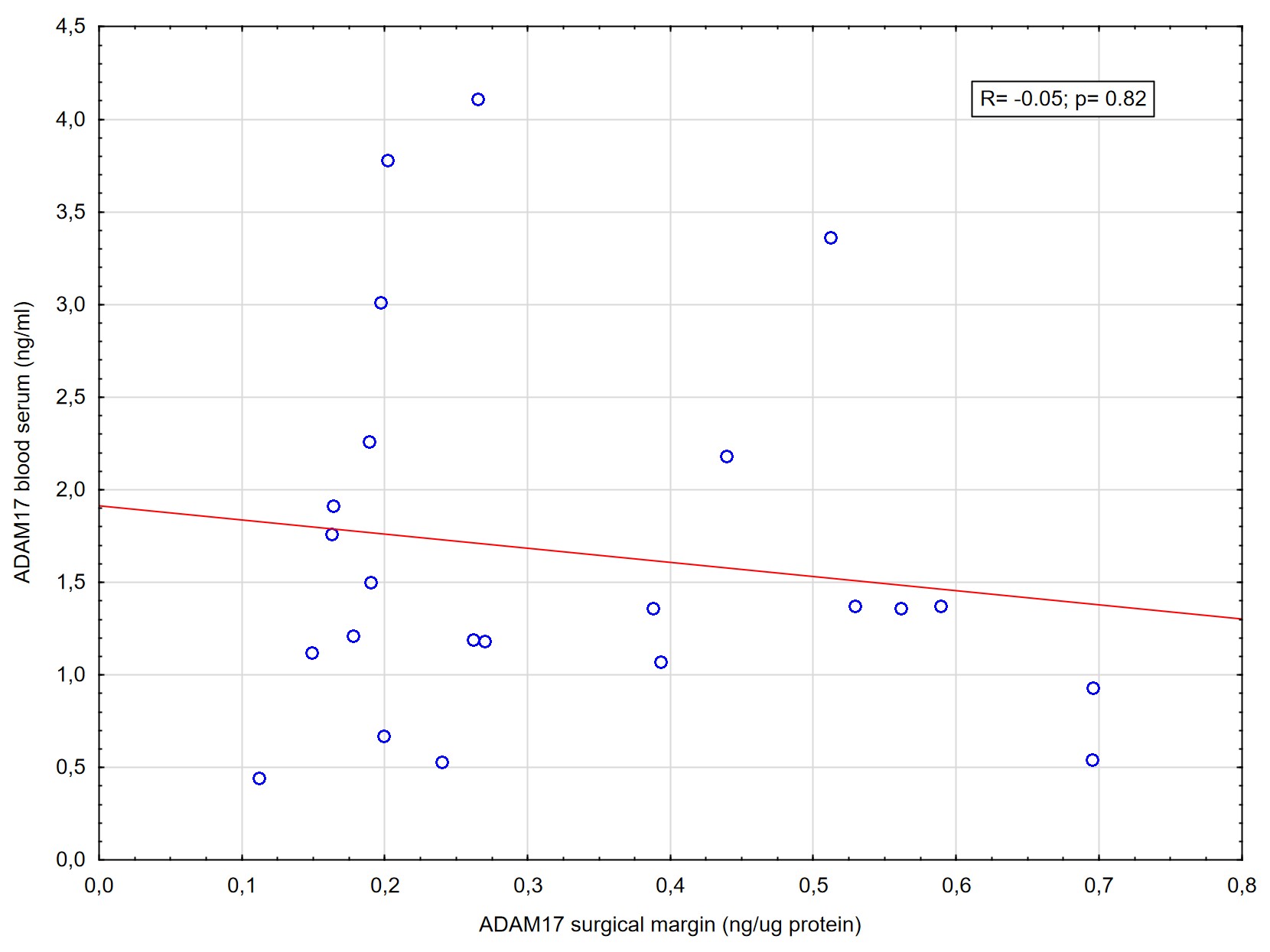

Supplement: Supplementary file 1 [file ijms-26-01104-s001.zip › Figure S11. Correlation between ADAM17 concentration in surgical margin tissue and ADAM17 concentration in blood serum..jpg]

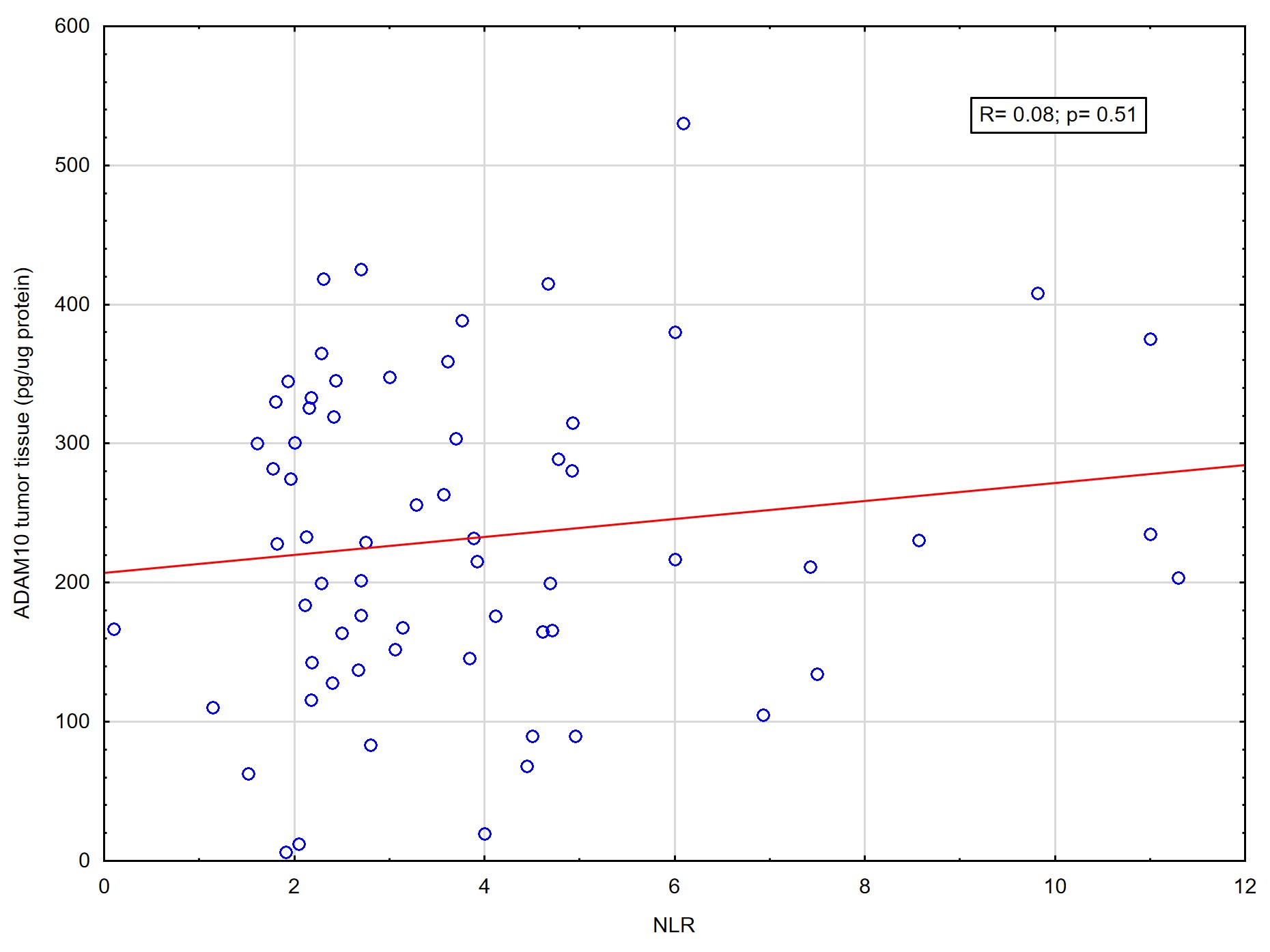

Supplement: Supplementary file 1 [file ijms-26-01104-s001.zip › Figure S1. Correlation between NLR and ADAM10 concentration in tumor tissue..jpg]

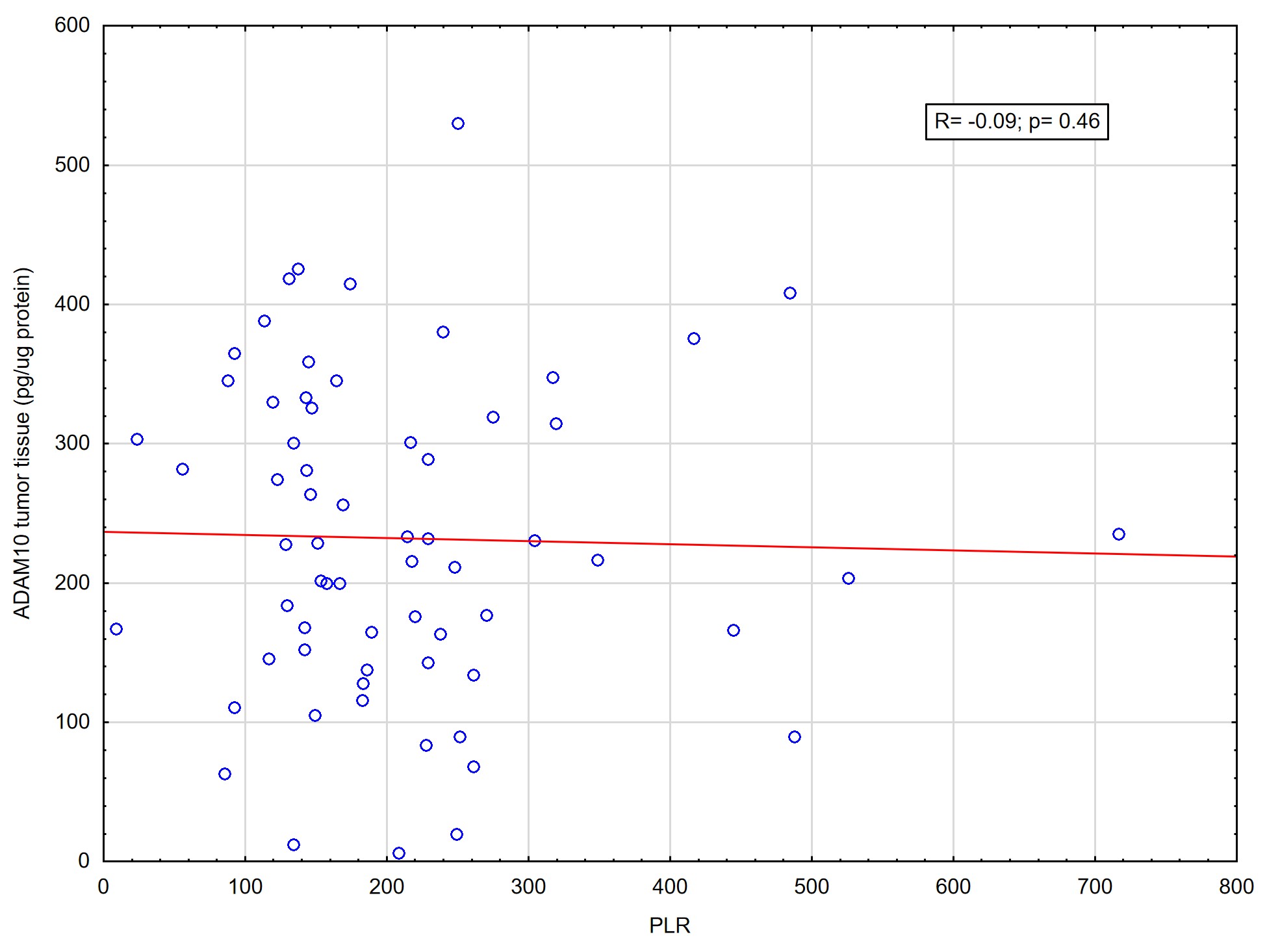

Supplement: Supplementary file 1 [file ijms-26-01104-s001.zip › Figure S2. Correlation between PLR and ADAM10 concentration in tumor tissue..jpg]
